# Gender differences in eating habits and lifestyles of young students: observational evaluation from MaestraNatura program

**DOI:** 10.3389/fnut.2025.1653154

**Published:** 2025-11-19

**Authors:** Annalisa Silenzi, Letizia Giona, Giulia Zanchi, Alessia Tammaro, Valentina Monteleone, Massimo D’Archivio, Carmela Santangelo, Roberta Masella, Beatrice Scazzocchio, Rosaria Varì

**Affiliations:** 1 Center for Gender-Specific Medicine, Istituto Superiore di Sanità, Rome, Italy; 2 Center for Behavioral Sciences and Mental Health, Istituto Superiore di Sanità, Rome, Italy; 3 Faculty of Medicine and Surgery, Catholic University of the Sacred Heart, Rome, Italy; 4 Department of Biomedicine and Prevention, University of Rome “Tor Vergata”, Rome, Italy

**Keywords:** eating habits, lifestyle, education, school, students, gender differences

## Abstract

**Introduction:**

Unhealthy diet and sedentary life represent the main risk factors for chronic NonCommunicable Diseases. Investing in children’s health education leads to benefits for their whole lifespan and the whole of society. MaestraNatura (MN) is an education program addressed to increase knowledge and skills about food and nutrition in students from primary and first-level secondary school. The main objective of the present paper was to evaluate eating habits and the adherence to the principles of the Italian dietary guidelines (IDG) of 8–14 years old children to identify possible influences of gender and parents on their eating choices and behaviors. Therefore, the potential benefits of MN on promoting changes in lifestyle and dietary patterns were also evaluated.

**Methods:**

Questionnaires on eating habits and physical activity were completed by 1,037 students and 220 parents from primary and secondary schools from 6 Italian regions. One hundred fifty-eight questionnaires were filled in by a subgroup of secondary schools’ students before and after two consecutive years of MN program. Differences between groups in the responses to questions were assessed by Chi-squared (*χ*^2^) tests. Differences in the IDG adherence score were assessed by analysis of variance (ANOVA), with gender (males, M vs. females, F) and/or type of school as between-subjects’ factors, as appropriate.

**Results:**

The questionnaires showed an average degree of adherence to the principles of the IDG for children with a similar distribution in M and F. Analysis of individual responses revealed differences between F and M in daily consumption of vegetables (F > M), water (M > F), and in weekly consumption of nuts (F > M) and commercial cookies (M > F). Furthermore, M are more engaged in physical exercise compared to F. Results from a subgroup of students attending MN for two consecutive years showed increases in the consumption of water, vegetables, fruit and legumes, and a decrease in the intake of commercial snacks, as well as an increase in physical activity.

**Conclusion:**

Italian children evidence gender differences in eating behaviors. Specific nutrition programs are needed to raise children’s awareness of the importance of a healthy lifestyle and to correct eating habits.

## Introduction

Inappropriate lifestyle behaviors, such as tobacco smoking, inadequate diet, excessive alcohol consumption, and sedentary lifestyle represent the main risk factors for chronic Non-Communicable Diseases (NCDs) (1). An adequate and balanced dietary pattern, made from evidence-based indications provided by the scientific community and based on the principles of Mediterranean Diet (MD) plays a protective and/or preventive role against specific pathological conditions (2). The 2024 World Health Organization (WHO)‘s report on Obesity and overweight revealed that in 2022, 37 million children under the age of 5 were overweight and over 390 million children and adolescents aged 5–19 years were overweight, including 160 million who were living with obesity (3). Moreover, physical inactivity represents a cause of concern too, given that in the European region only 25% of boys and 15% of girls achieve 60 min of physical exercise daily as recommended by WHO for young people (4).

The 2025 WHO global nutrition targets for maternal, infant and young child nutrition provide to preventing increase in childhood overweight (5). It is worth of note that the WHO, in the 2030 Agenda for Sustainable Development (6), included as goal 3 the reduction of premature deaths from NCDs through prevention and treatment as well as by promoting well-being. Moreover, the goal 4 aims to “*ensure that all learners acquire the knowledge and skills needed to promote sustainable development, including, among others, through education for sustainable development and sustainable lifestyles*.”

Community-based interventions carried out in school and early childcare settings have been identified as key strategies for health promotion by influencing the adolescents’ dietary habits and engaging families as well. Teaching children and their families to recognize appropriate portion sizes and to make conscious food choices is crucial for the success of any dietary strategy for the prevention of metabolic diseases (7). Food consumption preferences are developed early in life, therefore a life course approach addressed to increase knowledge together with skills that support healthy behaviors should begin as early as possible and continue longitudinally through childhood, adolescence, and young adulthood, with transition into adult care (8).

However, some studies have shown that there is no direct correlation between the information received (knowledge acquired) and the response of individuals (9, 10). This can be attributed to the presence of a much more complex and multifaceted set of barriers, which depend on external factors (e.g., family, socio-economic, cultural environment), most of them related to gender. Gender has been recognized as an important factor influencing lifestyle habits and, consequently, the onset and course of chronic diseases (11). At the same time, differences due to gender are closely linked to those due to sex since each of them has a strong impact on eating habits and individual response to food intake (12). Previous research has shown how gender can influence food choices and tastes (13, 14). Men and women exhibit significant differences in reward circuits and neural responses to food stimuli, which may underlie distinct patterns of food consumption (15). Examining the contribution of gender in eating habits, food preferences, and nutritional knowledge could offer crucial insights into the design of more effective and personalized nutritional interventions (16). Most studies to date have examined gender differences in food-related behaviors among adults, but, to the best of our knowledge, little attention has been given to these factors in children. Consequently, it remains unclear whether and when the influence of gender on food choices starts to be relevant.

This study aims to explore eating habits and adherence to the Italian Dietary Guidelines (IDG) (17) among children aged 8 to 14 enrolled in the MaestraNatura (MN) program, an innovative nutrition education initiative developed by the Istituto Superiore di Sanità (18, 19) to identify possible influences of gender and parents on their dietary choices and behaviors. Additionally, the study seeks to evaluate the impact of the MN program on promoting lifestyle changes among a subgroup of students who participated in the program’s educational activities for two consecutive years, with a particular focus on identifying possible gender differences.

## Methods

### Ethical aspects

Parents signed the informed consent to allow the participation of their children in the MN program as required by the Italian law regarding ethical and legal (personal data protection) aspects. The objectives of the study and the required activities were explained to teachers and parents in meetings and leaflets before the start of the study. Participation in the program was on a voluntary basis. The study was approved by the ethics committee of Istituto Superiore di Sanità (AOO-ISS 26.04.21 n.0015951) (18).

### Study participants

**Figure 1:**
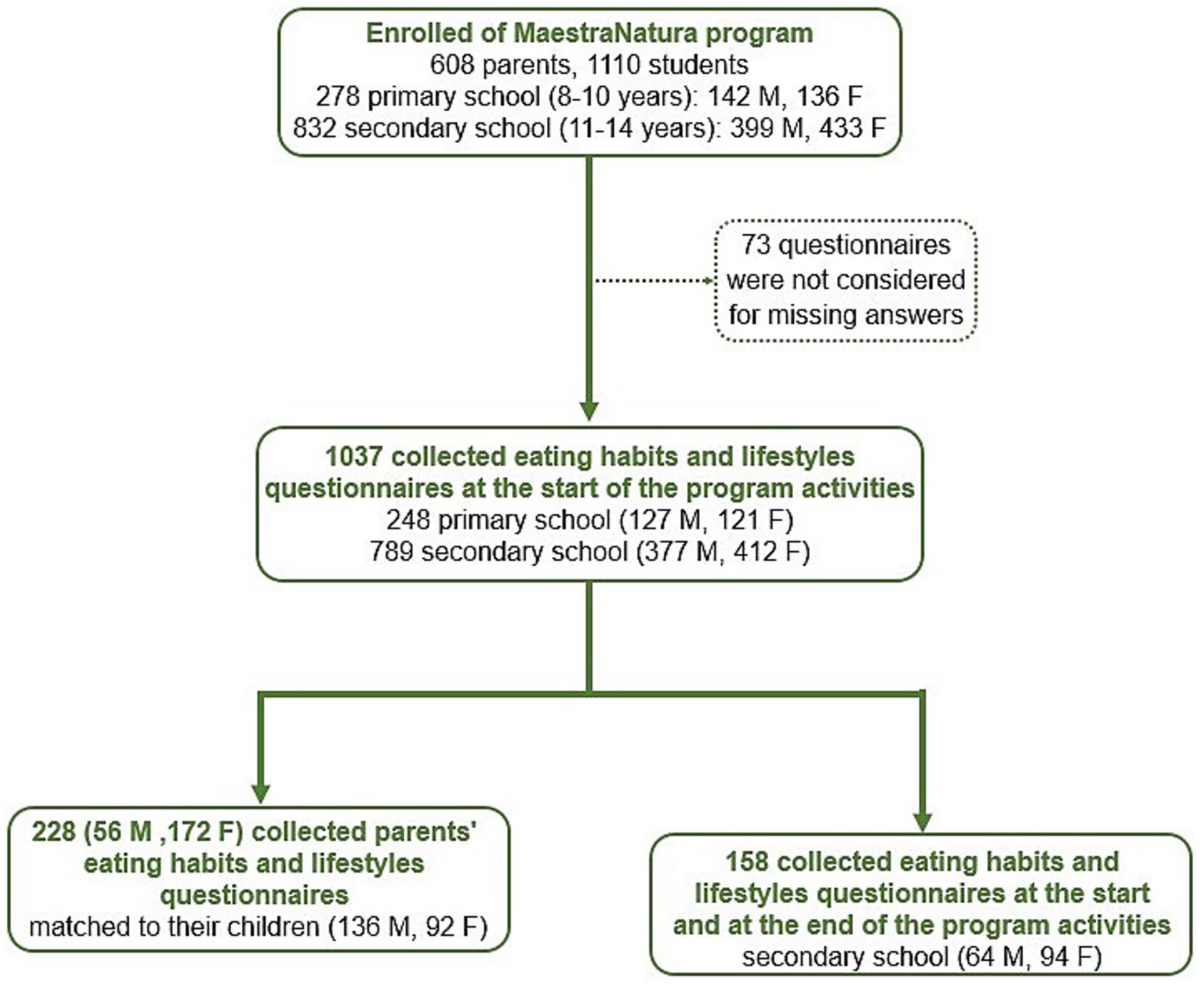
Flow chart of the study and subjects’ recruitment. F, female; M, male.

**Figure 2:**
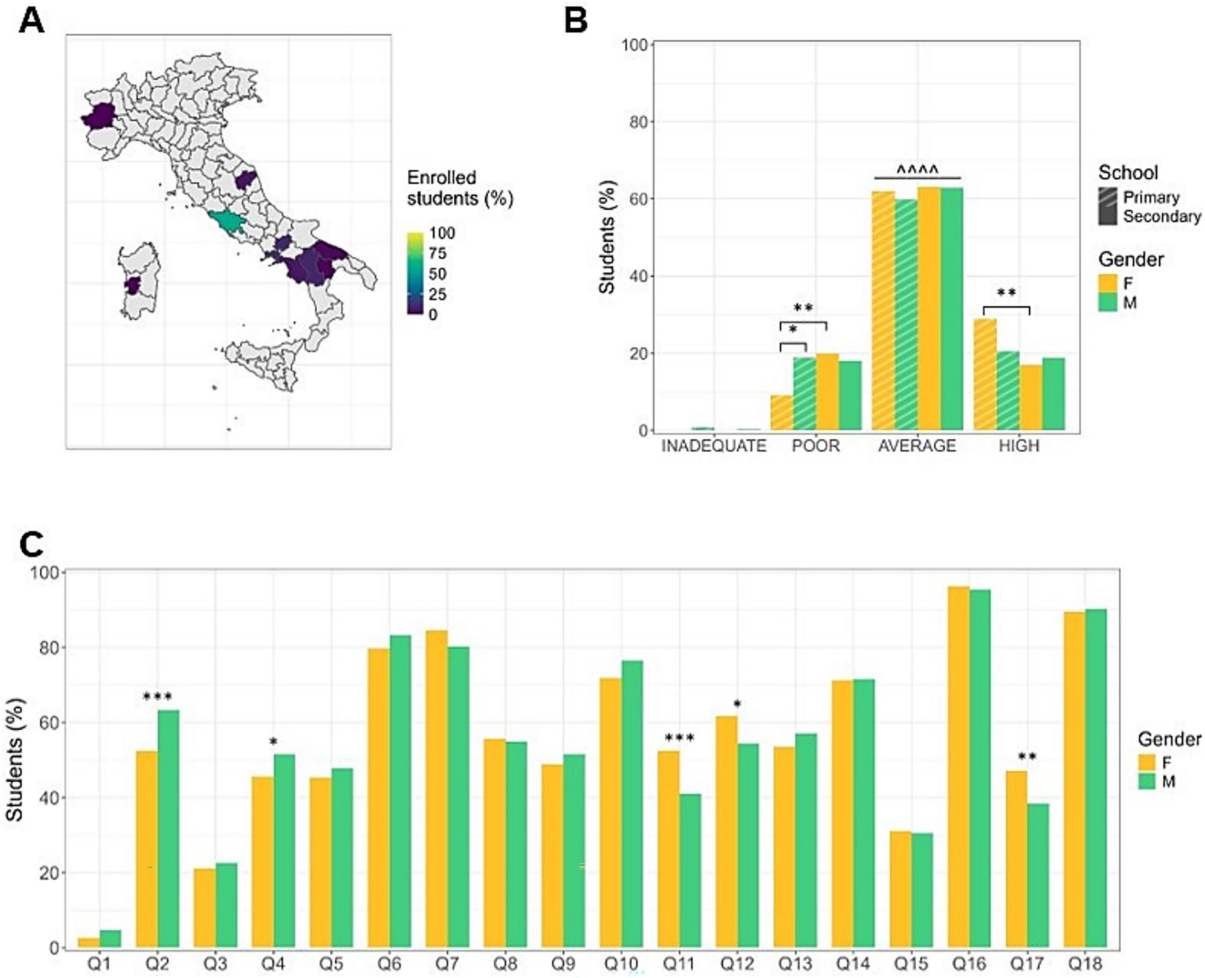
Adherence to the principles of the Italian Dietary Guidelines (IDG) by girls and boys. **(A)** Territorial distribution of participants with percentage of enrolled students per city. **(B)** Level of adherence to the principles of IDG by educational stages and gender of the enrolled students. Sixty percent of students show an average level of adherence, with younger females proving to be more virtuous. Data are expressed as frequencies. ^^^^ *p* < 0.0001 average vs. inadequate, poor, high; * *p* < 0.05, ** *p* < 0.01. **(C)** Gender differences in eating habits. Females show a higher consumption of vegetables and dried fruits, while males drink more water and eat more commercial cookies. Data are expressed as frequencies. * *p* < 0.05, ** *p* < 0.01, *** *p* < 0.001. F: Female; M: Male; Q: Question.

1,110 students from primary (8–10 years) and first-level secondary (11–14 years) public schools and 608 parents were enrolled in the MN program (18–20). The schools were mainly located in the center and south of Italy: 278 students attended primary school (142 boys and 136 girls) and 832 secondary school (399 boys and 433 girls) (Figures 1, 2A). In each school, MN group was organized so that the socio-cultural and economic characteristics of the students were as homogeneous as possible. The methodological approach was built upon the active participation of teachers and students according to previous research from our group (18–20).

### Evaluation of dietary habits and physical activity of students and parents

To estimate dietary habits of the students, an 18-item questionnaire was administered (21) (Table 1). IDG score was calculated by assigning 1 point to the positive answers and 0 points to the negative ones. On the basis of the score obtained by each participant the subjects were assigned to four categories indicative of different degrees of adherence to the Italian guidelines for a healthy diet (inadequate ≤ 4, poor 5–9, average 10–13 and high ≥ 14–18 points). Moreover, all the participants were requested to indicate the number of days per week on which they were engaged in physical activity (PA) (at least 60 min a day of moderate to vigorous activity, including outdoor play and formal sports activities) (22): “No day/week,” “1–3 times/week” or “Daily.”

**Table 1:** Students’ questionnaire on dietary habits.

Question number	Question
Q1	Do you go to a fast-food restaurant more than once a week?
Q2	Do you drink at least 1.5 liters of water a day?
Q3	Do you drink carbonated and sugary drinks (e.g., Cola)?
Q4	Do you eat commercial sweets (e.g., croissants, snacks)?
Q5	Do you consume milk and/or yogurt at least twice a day?
Q6	Do you eat breakfast at least 5 days a week?
Q7	Do you eat legumes at least once a week?
Q8	Do you eat legumes more than once a week?
Q9	Do you eat fish at least twice a week?
Q10	Do you eat at least 2–3 servings of cereals (e.g., pasta, rice, spelt)?
Q11	Do you eat raw or cooked vegetables more than once a day?
Q12	Do you eat a serving of raw or cooked vegetables every day?
Q13	Do you eat more than one fruit every day?
Q14	Do you eat a fruit, or a juice or a smoothie every day?
Q15	Do you eat sweets and candy several times a day?
Q16	Do you use extra-virgin olive oil?
Q17	Do you eat nuts (e.g., walnuts, almonds, pistachios)?
Q18	Do you eat cereals or baked goods (e.g., bread, cookies)?

To compare the dietary and physical activity habits of parents and children, parents were given a 26-item multiple-choice questionnaire modified by Turconi et al. (23) (Table 2), selecting questions that matched those administered to the children. IDG score was calculated by assigning five different scores for each answer ranging from −1 to 3.

**Table 2:** Parents’ questionnaire on eating habits and lifestyle.

Question number	Question
Q1	How many servings of raw or cooked vegetables do you consume per day?
Q2	How many servings of fresh fruit do you consume on average per day?
Q3	How many servings of cereals and tubers do you consume on average per day?
Q4	How many tablespoons of oil (large: 13 g) do you consume on average per day?
Q5	How many servings of milk or yogurt do you consume on average per day?
Q6	How many servings of legumes do you consume on average per week?
Q7	How many servings of fresh fish, mollusks, or shellfish do you consume per week?
Q8	How many servings of fresh or frozen red meat do you consume per week?
Q9	How many servings of fresh or frozen white meat do you consume per week?
Q10	How many servings of processed meats do you consume on average per week?
Q11	How many eggs do you consume on average per week?
Q12	How many servings of fresh and aged cheeses do you consume per week?
Q13	How many servings of nuts do you consume on average per week?
Q14	Do you regularly consume (2–3 times a week) butter or margarine?
Q15	How many days per week do you eat breakfast?
Q16	How many days per week do you eat meals outside the home on average?
Q17	How many times per week do you eat at fast food on average?
Q18	How many times per week do you consume wine or beer on average?
Q19	How many days per week do you engage in physical activity for 30 min on average?
Q20	How many times per week do you consume carbonated drinks on average?
Q21	Do you use extra virgin olive oil as main fat?
Q22	How many times per week do you consume bitters, grappa, or similar spirits on average?
Q23	How much water do you consume on average per day?
Q24	How many days per week do you engage in physical activity for 60 min on average?
Q25	How many days per week do you consume pre-cooked or ready-to eat foods on average?
Q26	How many times per week do you consume pastry products on average?

### Evaluation of the impact of MN program in inducing changes in eating behavior

To assess possible changes in eating behavior and lifestyles patterns following MN program, a subgroup of 158 students (64 boys and 94 girls from first-level secondary school) attending the MaestraNatura program was monitored for two consecutive years and was requested to fill out the lifestyles questionnaire (Table 1) at the start (T0) and the end (T1) of the two-year didactic path. All the students took part in the theoretical and practical activities planned by the MN educational paths provided for each class. Each path included PowerPoint presentations, exercises and experiments aimed at increasing nutrition knowledge, thus promoting awareness of the importance of a balanced and varied diet (20). In addition, the learning path included a “how to cook” section, reporting recipes to cook at home together with parents to promote interaction between them and encourage children to taste new food, especially vegetables. The MN didactic activities spanned the entire school year. It was possible to download all the contents from the MN web platform, which is divided into different areas specifically addressed to teachers, parents, and students.^1^

### Statistical analysis

The dataset analyzed for the evaluation of dietary habits and physical activity included answers from 1,037 students grouped as follow: 248 students from primary school (127 boys and 121 girls) and 789 from secondary school (377 boys and 412 girls). Cronbach’s alphas (0.54–0.66) were computed to measure the internal consistency of the students’ eating habits questionnaire. An IDG adherence score was generated assigning 0 to 18 points deriving from correct answers to questions Q1–18 and differences in mean score values were assessed by analysis of variance (ANOVA), with gender male (M) vs. female (F) and/or type of school (Primary vs. Secondary) as between-subjects’ factors, as appropriate. The Tukey’s post-hoc test was used following ANOVA when a significant interaction was found. Then, to evaluate differences between groups in the responses of questionnaire item Q1-18, therefore differences for each specific eating habit, as well as in physical activity habits, Chi-squared (*χ*^2^) tests were used.

The subpopulation whose parents were involved in the study consisted of 228 students (136 boys and 92 girls) from both primary and secondary school. Linear regression models were used to assess the relationship between parents’ and their children’s IDG adherence while binary logistic regressions were performed to investigate the influence of specific parental dietary habits on those of their children, as well as physical activity habits. For the latter, the students’ response was converted to 0 when their answers were equal to “No day/week” or to 1 in the other cases, to obtain a binary classification comparable to those of the other questions.

The dataset analyzed for the evaluation of the impact of MN program in inducing changes in eating behavior, after 2 years of didactical path, included answers from 120 students from secondary school (42 boys and 78 girls). Also in this case, to evaluate differences for each specific eating/physical activity habit between genders as well as between before (T0) and after (T1) joining the MN program, responses of questionnaire item Q1-18 were analyzed by Chi-squared (*χ*^2^) tests.

A *p*-value < 0.05 was considered statistically significant. The Bonferroni’s correction was applied to take into account the increase in Type I error probability due to multiple tests, where appropriate, and both the original and adjusted *p*-values were reported. Effect sizes were measured for each statistical comparison to quantify the magnitude of significant differences. In particular, the Phi coefficient (*φ*) for Chi-squared tests, Cohen’s d (d) for ANOVA, R squared (*R*^2^) coefficient for linear regressions and Odds ratios (OR) for logistic regressions were reported. Data are expressed as frequencies or as mean ± standard error of the mean (SEM), as appropriate. All analyses and plots were performed using R version 4.2.2 (R Software for Statistical Computing, Vienna, Austria).

## Results

### Evaluation of the adherence to Italian dietary guidelines

1,037 questionnaires were collected to evaluate eating habits and the adherence to the principles of the IDG from Italian primary and first-level secondary school students (Figure 2A). Sixty percent of the students demonstrated an average level of adherence to the principles of IDG with a similar distribution among M and F (Figure 2B). Specifically, about 60% of the students consume fruit every day, about 50% eat vegetables once a day, about 45% consume milk/yogurt at least twice a day (Figure 2C).

However, considering individual questions, data disaggregated by sex revealed significant differences in some eating behaviors. Notably, significant differences were found between F and M in the daily consumption of vegetables (F > M) (Q11: *χ*^2^ = 13.66, *p* = 0.0002, *φ* = 0.11; Q12: *χ*^2^ = 5.77, *p* = 0.0163, φ = 0.07) and water (M > F) (Q2: *χ*^2^ = 12.29, *p* = 0.0005, φ = 0.10), and in the weekly consumption of nuts (F > M) (Q17: *χ*^2^ = 7.82, *p* = 0.0052, *φ* = 0.08) and commercial cookies (M > F) (Q4: *χ*^2^ = 3.73, *p* = 0.0535, φ = 0.05; Figure 2C).

### Differences in eating habits in students from primary and secondary school

**Figure 3:**
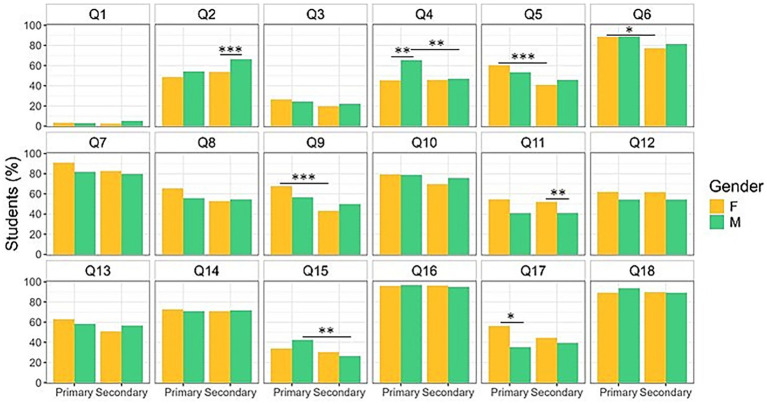
Eating habits by educational stages and gender of the enrolled students. Older females consume more vegetables compared to age-matched males; by contrast, older males drink more water. Moreover, younger females consume more yogurt and fish than older females, while younger males eat more snacks and candies compared to both same age females and older males. Data are expressed as frequencies. * *p* < 0.05, ** *p* < 0.01, *** *p* < 0.001, **** *p* < 0.0001. Q: Question.

The analysis of data stratified by age showed statistically significant variations between primary and secondary educational stages. In general, the younger student cohort exhibits significantly higher compliance with the IDG compared to the older cohort [*F* (1,1,035) = 3.9838, *p* = 0.0462, d = 0.15] with a significant difference observed in the high adherence scores (*χ*^2^ = 5.44, *p* = 0.0197, *φ* = 0.06) (data not shown). When stratifying the data by individual responses, it appears that younger students demonstrate more virtuous behaviors than older students. Specifically, primary school students have breakfast every day (Q6: *χ*^2^ = 11.28, *p* = 0.0008, *φ* = 0.10), eat fish weekly (Q9: *χ*^2^ = 18.33, *p* < 0.0001, φ = 0.13), consume yogurt and/or milk 2–3 times a day (Q5: *χ*^2^ = 13.84, *p* = 0.0002, φ = 0.11), eat cereals and pasta daily (Q10: *χ*^2^ = 4.04, *p* = 0.0443, φ = 0.05) (Figure 3). Conversely, younger students consume less water (Q2: *χ*^2^ = 5.05, *p* = 0.0246, φ = 0.06) and have a higher intake of commercial cookies (Q4: *χ*^2^ = 6.65, *p* = 0.0099, *φ* = 0.07), sweets and candies (Q15: *χ*^2^ = 8.48, *p* = 0.0036, *φ* = 0.08) compared to older students (Figure 3).

#### Sex and gender differences

**Figure 4:**
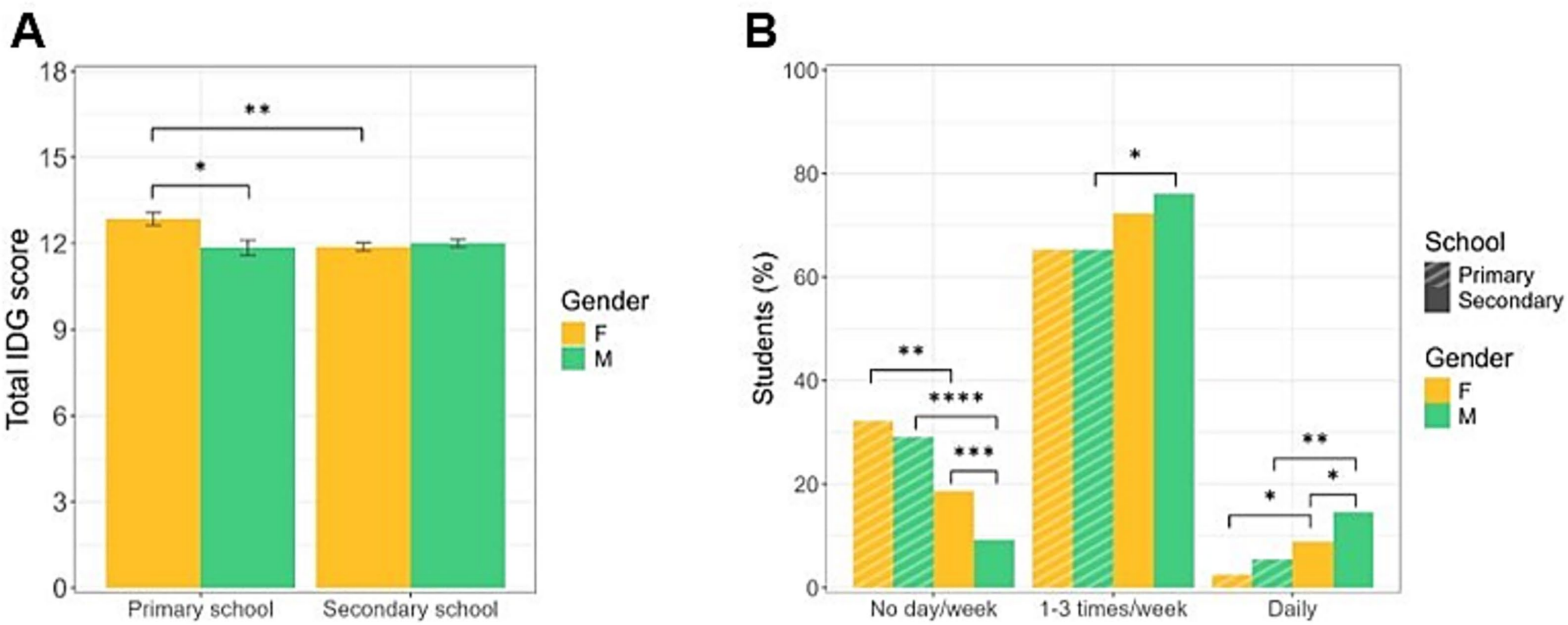
Adherence to IDG and physical activity habits. **(A)** Mean total scores of adherence to the principles of IDG by educational stages and gender of the enrolled students. Primary school females show higher scores. Data are expressed as mean ± SEM. * *p* < 0.05, ** *p* < 0.01. **(B)** Physical activity habits by educational stages and gender of the enrolled students. Older students engage in more physical activity, with secondary school males being the most active. Data are expressed as frequencies. * *p* < 0.05, ** *p* < 0.01, *** *p* < 0.001, **** *p* < 0.0001.

Furthermore, when disaggregated by age and sex, data show that primary school females achieved higher scores compared to both primary school males and secondary school females [*F* (1,1,033) = 8.081, *p* = 0.0046, d = 0.36; F-primary vs. M-primary: *p* < 0.05, F-primary vs. F-secondary: *p* < 0.01] (Figure 4A). Notably, primary school males consume more commercial cookies than females (Q4: *χ*^2^ = 9.94, *p* = 0.0016, P-adj = 0.0064, *φ* = 0.19). Females in secondary school eat more vegetables (Q11: *χ*^2^ = 9.27, *p* = 0.0023, P-adj = 0.0092, *φ* = 0.18) than their male peers in the same age groups; additionally, younger females consume more nuts (Q17: *χ*^2^ = 10.77, *p* = 0.0010, P-adj = 0.0040 *φ* = 0.20) than males from the same cohort. In contrast, secondary school males drink more water than age-matched females (Q2: *χ*^2^ = 13.14, *p* = 0.0003, P-adj = 0.0012, *φ* = 0.12; Figure 3; Supplementary Table S1).

Notably, the higher intake of commercial cookies, sweets and candies observed in primary school students, described above, can be ascribed to males only (younger M > older M, Q4: *χ*^2^ = 12.88, *p* = 0.0003, *p*-adj = 0.0012, φ = −0.16; Q15: *χ*^2^ = 11.45, *p* = 0.0007, P-adj = 0.0028, φ = −0.15). By contrast, the consumption of yogurt and/or milk 2–3 times a day, breakfast every day as well as eating weekly fish is due to females only (younger F > older F, Q5: *χ*^2^ = 14.07, *p* = 0.0002, *p*-adj = 0.0008, *φ* = 0.16; Q6: *χ*^2^ = 7.32, *p* = 0.0068, P-adj = 0.0272, *φ* = 0.12; Q9: *χ*^2^ = 22.14, *p* < 0.0001, *p*-adj < 0.0001, φ = 0.20; Figure 3; Supplementary Table S1).

### Assessment of physical activity of the students

Data on physical activity habits indicate that approximately 20% of the students are inactive (Figure 4B). Specifically, primary school students engage in less physical activity compared to older students. In particular, a higher percentage of younger students—approximately 30%—report not participating in any physical activity, compared to their secondary school counterparts (*χ*^2^ = 34.40, *p* < 0.0001, *φ* = 0.19); moreover, primary school students do less physical activity both 1–3 days a week or daily (weekly activity: χ^2^ = 7.29, *p* = 0.0069, *φ* = 0.08; daily activity: *χ*^2^ = 12.38, *p* = 0.0004, φ = 0.10). Interestingly, younger females are, in general, less active (No day/week) compared to older students (*χ*^2^ = 10.07, *p* = 0.0015, *p*-adj = 0.0060, φ = 0.13); a significantly higher proportion of older females report not be engaged in physical activity compared to males in the same age group (*χ*^2^ = 14.30, *p* = 0.0002, *p*-adj = 0.0008, φ = 0.13). Furthermore, analysis of groups doing physical exercise 1–3 days a week or daily reveals that secondary school males demonstrated significantly higher participation in getting active compared to younger males (daily activity: *χ*^2^ = 7.26, *p* = 0.0071, *p*-adj = 0.0284 φ = 0.11) and age-matched females (daily activity: *χ*^2^ = 6.01, *p* = 0.0142, *p*-adj = 0.0568, φ = 0.08; Figure 4B; Supplementary Table S2).

### Evaluation of the influence of the parents on students’ lifestyles

**Figure 5:**
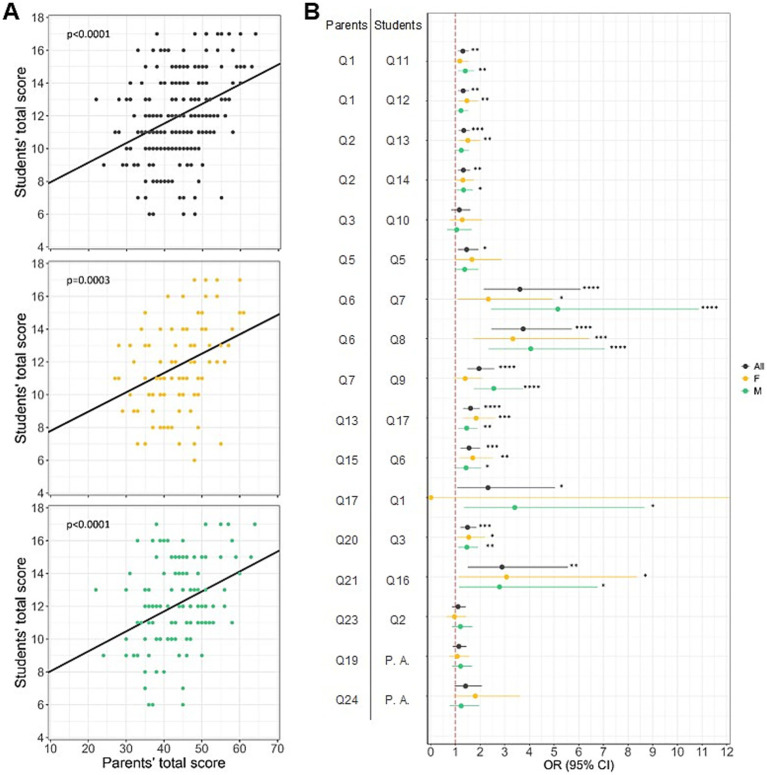
Influence of the family social context on the habits and lifestyles of children. **(A)** Relationship between parents’ and students’ total scores of adherence to the principles of IDG. Linear regressions showing that students’ total scores are positively related with parents’ total scores, regardless of gender, indicating that both girls (plot with orange points) and boys (plot with green points) are influenced by their family’s eating habits. **(B)** Relationship between parents’ and students’ specific eating habits. Logistic regression results showing that, overall, students follow their parents’ behavior, except for water and cereals/tubers consumption. Gender differences can be observed in fish and fast-food consumption, with males mirroring their parents’ behavior. Dots represent the odds ratio for each question match (parents’ habits and their children’s respective habits); horizontal lines indicate the 95% confidence intervals; vertical red dashed lines represent the null association threshold (i.e., if the confidence intervals for a question match overlap with this line, there is no association between the parents’ habit and the child’s habit). * *p* < 0.05, ** *p* < 0.01, *** *p* < 0.001, **** *p* < 0.0001. Q = Question; P. A.: Physical activity.

One of the purposes of our study was to compare students’ and parents’ lifestyles in order to identify the influence of the family social context on the habits and lifestyles of children. By analyzing in detail 228 parents’ lifestyles questionnaires matched to those filled out by their children, it was highlighted that overall children followed parents’ behaviors (overall: *F* (1,226) = 33.05, *p* < 0.0001, *R*^2^ = 0.13; females: *F* (1,90) = 13.76, *p* = 0.0003, *R*^2^ = 0.13; males: *F* (1,134) = 19.37, *p* < 0.0001, *R*^2^ = 0.13; Figures 5A,B), except for water and cereals/tubers consumption (Q23-Q2: *p* > 0.05, OR = 1.10, 95% CI = 0.86–1.42, Q3-Q10: *p* > 0.05, OR = 1.16, 95% CI = 0.84–1.60; Figure 5B). Gender differences were also observed in children mirroring some parents’ eating behaviors; specifically, males consume regularly fish (twice a week) (Q7-Q9: *p* < 0.0001, OR = 2.55, 95% CI = 1.74–3.75) and do not go to the fast-food restaurant (Q17-Q1: *p* < 0.05, OR = 3.41, 95% CI = 1.34–8.67), while females, on the contrary, regarding to going to fast-food, behave in opposite manner with respect to their parents (Figure 5B).

### Assessment of the influence of MaestraNatura program in promoting healthy eating habits

By examining a subpopulation of students who participated in the MaestraNatura educational program for two consecutive years, we observed progress in healthy eating behaviors. Specifically, there was an increase in the consumption of water (Q2) and vegetables (Q11), primarily attributable to females (Q2: *χ*^2^ = 5.65, *p* = 0.0174, *p*-adj = 0.0696, *φ* = 0.17; Q11: *χ*^2^ = 5.85, *p* = 0.0156, *p*-adj = 0.0624, φ = 0.18). Additionally, both sexes showed a positive trend toward increased fruit consumption (Q13, Q14), while fish intake (Q9) increased mainly among females, reaching the higher levels of the males. Notably, males demonstrated a reduction in snack consumption (Q4), ultimately aligning with the lower levels observed in females by the end of the program (Table 3). On the contrary, some behaviors were worsened as well as going to fast-food restaurant more than once a week (Q1) and having breakfast regularly (Q6). Specifically, females go fast-food restaurants more frequently even though overall the percentage remains under 5%; moreover, both sexes show a worsening in daily breakfast consumption, about 75% maintains regular breakfast habits (Table 3).

**Table 3:** Proportion of students who answer affirmatively to the questions of each item of the IDG questionnaire before and after attending the MaestraNatura program.

Question	Gender	Proportion (%)		*p*-value	*p*-adj
		T0	T1		
Q1	F	1,28	8,97	0.0294*	0.1176
	M	2,38	4,76	0.5566	>0.999
	*p*-value	0.6538	0.4034		
	*p*-adj	>0.999	>0.999		
Q2	F	57,69	75,64	0.0174*	0.0696^
	M	73,81	78,57	0.6084	>0.999
	*p*-value	0.0805^	0.7173		
	*p*-adj	0.3220	>0.999		
Q3	F	14,10	17,95	0.5126	>0.999
	M	21,43	19,05	0.7860	>0.999
	*p*-value	0.3044	0.8820		
	*p*-adj	>0.999	>0.999		
Q4	F	33,33	34,62	0.8658	>0.999
	M	57,14	40,48	0.1265	0.5060
	*p*-value	0.0116*	0.5251		
	*p*-adj	0.0464*	>0.999		
Q5	F	41,03	38,46	0.7435	>0.999
	M	42,86	47,62	0.6611	>0.999
	*p*-value	0.8461	0.3318		
	*p*-adj	>0.999	>0.999		
Q6	F	78,21	69,23	0.2029	0.8116
	M	90,48	78,57	0.1315	0.5260
	*p*-value	0.0915^	0.2744		
	*p*-adj	0.3660	>0.999		
Q7	F	82,05	84,62	0.6674	>0.999
	M	78,57	85,71	0.3927	>0.999
	*p*-value	0.6441	0.8723		
	*p*-adj	>0.999	>0.999		
Q8	F	50,00	53,85	0.6307	>0.999
	M	52,38	61,90	0.3778	>0.999
	*p*-value	0.8035	0.3955		
	*p*-adj	>0.999	>0.999		
Q9	F	42,31	51,28	0.2613	>0.999
	M	61,90	57,14	0.6566	>0.999
	*p*-value	0.0405*	0.5393		
	*p*-adj	0.1620	>0.999		
Q10	F	74,36	70,51	0.5909	>0.999
	M	78,57	71,43	0.4497	>0.999
	*p*-value	0.6072	0.9162		
	*p*-adj	>0.999	>0.999		
Q11	F	46,15	65,38	0.0156*	0.0624^
	M	35,71	45,24	0.3739	>0.999
	*p*-value	0.2699	0.0327*		
	*p*-adj	>0.999	0.1308		
Q12	F	71,79	69,23	0.7255	>0.999
	M	54,76	54,76	>0.999	>0.999
	*p*-value	0.0606^	0.1149		
	*p*-adj	0.2424	0.4596		
Q13	F	43,59	53,85	0.2000	0.8000
	M	54,76	61,90	0.5067	>0.999
	*p*-value	0.2424	0.3955		
	*p*-adj	0.9696	>0.999		
Q14	F	66,67	67,95	0.8645	>0.999
	M	69,05	73,81	0.6291	>0.999
	*p*-value	0.7905	0.5040		
	*P*-adj	>0.999	>0.999		
Q15	F	20,51	24,36	0.5648	>0.999
	M	19,05	23,81	0.5949	>0.999
	*p*-value	0.8482	0.9465		
	*p*-adj	>0.999	>0.999		
Q16	F	96,15	93,59	0.4679	>0.999
	M	95,24	97,62	0.5566	>0.999
	*p*-value	0.8108	0.3341		
	*p*-adj	>0.999	>0.999		
Q17	F	48,72	48,72	>0.999	>0.999
	M	28,57	28,57	>0.999	>0.999
	*p*-value	0.0327*	0.0327*		
	*p*-adj	0.1308	0.1308		
Q18	F	88,46	82,05	0.2588	>0.999
	M	95,24	85,71	0.1371	0.5484
	*p*-value	0.2198	0.6076		
	*p*-adj	0.8792	>0.999		

*p < 0.05; ^*p* < 0.1.

#### Physical activity

The results collected on physical activity questionnaires before and after 2 years of the educational path highlight differences on physical exercise performed daily; this change was mainly due to males (*χ*^2^ = 3.98, *p* = 0.0461, *p*-adj = 0.1844, *φ* = 0.19). Moreover, females, who were not engaged in physical activity, decrease compared to the beginning of the path, (Table 4). Notably, a difference between males and females is also observable at the end of the didactic path (*χ*^2^ = 5.20, *p* = 0.0226, *p*-adj = 0.1130, φ = 0.19).

**Table 4:** Proportion of students who reported to do physical activity before and after attending the MaestraNatura program.

Physical activity frequency	Gender	Proportion (%)		*p*-value	*p*-adj
		T0	T1		
No day/week	F	14,10	7,69	0.1989	0.7956
	M	7,14	7,14	>0.999	>0.999
	*p*-value	0.2573	0.9132		
	*p*-adj	>0.999	>0.999		
1–3 times/week	F	79,49	82,05	0.6845	>0.999
	M	83,33	66,67	0.0778^	0.3112
	*p*-value	0.6097	0.0574^		
	*p*-adj	>0.999	0.2870		
Daily	F	6,41	10,26	0.3848	>0.999
	M	9,52	26,19	0.0461*	0.1844
	*p*-value	0.5368	0.0226*		
	*p*-adj	>0.999	0.1130		

*p < 0.05; ^*p* < 0.1.

## Discussion

Promoting healthy diet, mainly in youth, through effective prevention programs, is the best way to fight unhealthy lifestyles, such as physical inactivity, dietary excesses and nutrition imbalances (24). Sex/gender related factors influencing lifestyle and exposition to risk factors seem to play major roles in the rising prevalence of obesity (25–27). In particular, men and women may be differently exposed to nutritional risk factors (11). Several studies carried out in adults have shown, indeed, significant differences in food preferences and dietary behaviors between men and women (28). On the contrary, very few data are available to define whether such differences exist also between young girls and boys. Our results showed an average degree of adherence to the Italian dietary guidelines in children revealing interesting differences between girls and boys in the daily consumption of vegetables (F > M), water (M > F) and in the weekly consumption of dried fruit (F > M) and commercial cookies (M > F), congruent with other studies demonstrating women are more prone to consume fruit and vegetables than men (14). Children from primary school generally spend half of their daily time within the school environment (29) and schools together with family are increasingly recognized as a pivotal factor in shaping young people’s dietary habits (30). The dietary habits of primary school children are predominantly influenced by the behaviors and decision making of their parents (30). However, the transition to secondary school brings substantial shifts in both the social and physical environment (31, 32). This period is marked by greater autonomy in food purchasing decisions, which are often influenced by peers, increased access to diverse food options, and heightened exposure to social media and advertisements promoting unhealthy foods (31–33). These findings are in line with our study that demonstrated younger students have more virtuous eating behaviors with respect to older ones, being most influenced by the dietary habits and lifestyles of the parents. Specifically, they tend to have breakfast every day, to eat fish weekly, consume regularly yogurt and/or milk, and eat cereals, pasta and nuts. The transition from childhood to adolescence is often characterized by changes in dietary habits, shifting toward less adequate dietary habits which makes adolescence a key time for public health intervention to improve students’ health (34). Thus, adolescence represents a pivotal developmental stage, making it a critical period for targeted nutritional interventions aimed at fostering awareness and promoting the adoption of healthy lifestyle habits. Effective and targeted public health interventions demand a multifaceted approach, addressing dietary patterns, physical activity, and sedentary behaviors, which often coexist and interrelate, shaping their role as key determinants of obesity (35). MaestraNatura is an education program developed and designed to meet this need. MN proposes a systemic-constructivist approach designed to enhance the understanding of complexity. This method simplifies the knowledge acquisition process without oversimplifying it and aims to effectively promote progressive, self-directed learning, which is characteristic of the constructivist approach. Additionally, it supports cooperative learning, a proven teaching strategy where small groups of students with varying abilities collaborate in diverse learning activities to deepen their comprehension of a subject (20). In previous studies, we have already demonstrated the effectiveness of the MN program in enhancing knowledge and skills about food and nutrition in students from primary (19) and first-level secondary school (18). In the present study, the lifestyles and the degree of adherence to the IDG of 11–14 years old children were assessed before and after two consecutive years of MN training intervention to evaluate its potential impact on raising awareness and promoting changes in eating habits and lifestyle. The data showed healthy and unhealthy eating habits changes, such as increased consumption of water, vegetables, fruit, and fish and a decrease in the consumption of commercial snacks, in addition to an increase in physical activity levels, providing further the importance of educational intervention in this crucial age to favor changes in lifestyle and dietary behaviors. The MN program influenced lifestyle changes differently in girls and boys. Specifically, it encouraged healthier behaviors in girls by increasing their consumption of water and vegetables, while in boys it promoted a reduction in snack intake. These findings emphasize the importance of adopting a gender-specific approach when designing educational interventions aimed at fostering healthier habits in children (36). An important point to highlight is the worsening of certain habits, such as the tendency to frequent fast-food restaurants (especially among girls) and skipping breakfast (in both sexes). This further emphasizes that the transition to adolescence is a critical period for intervention through targeted preventive strategies for both boys and girls, aimed at raising awareness and improving knowledge about the importance of a healthy diet for long-term well-being (31–33). Parents are important partners contributing indirectly to the success of school-based health promotion/preventive programs (37–39). Parents and family environment are crucial in the development of children’s dietary preferences that eventually lead to their dietary patterns (40). Our findings suggest that, overall, children tend to adopt their parents’ eating habits, even if the effect size is small. Indeed, only one-third of parents accurately completed the questionnaire on dietary habits and lifestyles, and only 30% of the participants were fathers. This may have introduced bias and contributed to the small effect size observed in the parental context. Therefore, it is essential to increase family involvement and promote prevention programs specifically targeting parents, particularly fathers, who are consistently underrepresented (41–43).

### Limitations

On the other hand, our study has some limitations. A significant limitation in assessing the beneficial effects of MN is the absence of a control group; consequently, we cannot rule out that the observed changes are due to adolescent autonomy and concurrent school activities. To strengthen the robustness of our findings, further studies including a control group and broader geographical representation are required. Furthermore, it would be valuable to conduct a follow-up evaluation of lifestyles, for example, 1 year after the study’s conclusion, to determine whether the observed changes are sustained over the long term.

## Conclusion

Early promotion of healthy eating is crucial to counteract unhealthy lifestyles and the growing prevalence of obesity. Our findings reveal gender differences in children’s dietary habits and the influence of parental behaviors in younger children. Adolescence emerges as a critical stage where dietary habits often decline, highlighting the need for focused, age-appropriate interventions. Overall, prevention strategies are key to fostering lasting healthy behaviors and reducing the risk of chronic diseases later in life. Consequently, policymakers must focus on developing and implementing nutrition policies and programs specifically designed for adolescents. These initiatives should carefully consider the distinct biological, social, and cultural factors that differently shape the dietary behaviors of boys and girls.

## Data Availability

The raw data supporting the conclusions of this article will be made available by the authors, without undue reservation.

## Footnotes

^1^ https://maestranatura.iss.it/#/app
